# Editorial: Infectious diseases and NCDs persist despite concerted effort

**DOI:** 10.4314/ahs.v21i2.1

**Published:** 2021-06

**Authors:** James K Tumwine

Welcome to this June 2021 issue of *African Health Sciences* which is rich in innovative research and practice papers from all over the world. We have purposely kept away from Covid-19 this season because it was distorting our perception of ill health in Africa and beyond.

Hence we have focused largely on infectious diseases. Our first paper comes from Egypt. El- Domany and colleagues^1^ report carbapenem resistant genes in Klebsiella pneumonia. It is followed by an interesting paper on genotypic detection of extended spectrum β-lactamases among *Escherichia coli* and *Klebsiella pneumoniae* isolates from type 2 diabetic patients with urinary tract infections keeping the *Klebsiella* theme alive^2^. Number 3 goes to: yes, *Klebsiella* again! It is to a paper on the prevalence and antimicrobial susceptibility of extended-spectrum beta lactamases-producing *Escherichia coli* and *Klebsiella pneumoniae* isolated in selected hospitals of Anyigba, Nigeria^3^. No runners up!

But our next paper is on ‘bacterial profile and their antimicrobial susceptibility patterns in patients admitted at Madda Walabu University Goba Referral Hospital, Ethiopia.’^4^. It is followed by a paper on brucellosis and its risk factors in humans and domestic ruminants in Kagera, Tanzania.^5^ Still in Tanzania, we have an interesting paper on bacterial vaginosis, the leading cause of genital discharge among women presenting with vaginal infection in Dar es Salaam.^6^

Asefa and others^7^ applied the Integrated Behavioral Model (IBM) to measure intention ‘to get early screening and treatment of Sexually Transmitted Infections (STIs) by HIV at- risk sub-populations' in Ethiopia. On the other hand, we have a report on unsafe sexual practices among truck drivers in India.^8^

This is followed by papers on urinary tract infections in Turkey^9^, oral health related quality of life among HIV positive patients in Nigeria^10^; and community pharmacists’ management of self-limiting infections^11^. We continue with papers on sexually transmitted infections among pregnant women in The Gambia^12^; experiences of sex workers accessing HIV care services in Zimbabwe^13^ and virologic suppression and associated factors in HIV infected Ugandan female sex workers^14^.

Then follows the prevalence and density of malaria parasitaemia amongst HIV individuals in Nigeria;^15^ and evaluation of postal service for referral of specimens of drug resistant tuberculosis in Ethiopia.^16^

Muzanyi and colleagues^17^ have written for us a paper on the ‘threat of persistent bacteria and fungi contamination in tuberculosis sputum cultures’ and we have a paper on factors associated with self-medication with antibiotics among University students in Tanzania.^18^ We end this treatise on infections with a paper on the role of body temperature on the respiratory rate in children with acute respiratory infections.^19^

Now to cancer. We have several oncology papers. First, Uganda authors^20^ have written on ‘detecting subclinical anthracycline therapy related cardiac dysfunction’; while Ghanaian workers bring to us theirs on ‘awareness and knowledge about prostate cancer among male teachers.’^21^ Then we have several papers on potential anticancer activity of on selected herbs^22^,^23^; followed by a case report^24^ on ‘transient bone marrow hypoplasia preceding T-Cell acute lymphoblastic leukemia.’ We end this section on cancer with a molecular study on nucleophosmin 1(NPM1) gene in acute myeloid leukemia^25^.

We continue this NCDs work with papers on diabetes mellitus. These include risk factors for type 2 diabetes mellitus in Nigeria^26^; and prediction of prevalence of type 2 diabetes in Rwanda.^27^ Kenyan scientists^28^ have written for us a seminal paper on ‘glycemic index values of traditional Kenyan foods.’ They contend that these values might be the missing link ‘in the effectiveness of dietary approach in the prevention and management of diabetes mellitus.’ Nigeria workers^29^, on the other hand, end this section on diabetes mellitus with a piece on ‘Streptozotocin-induced type 1 and 2 diabetes in rodents:’ a model for studying diabetic cardiac autonomic neuropathy.’

NCDs continue. We have papers on ‘circulatory microRNA expression profile for coronary artery calcification in chronic kidney disease patients;’^30^ and one on the ‘evaluation of three different laboratory methods to detect ‘human leukocyte antigen antibodies in a South African kidney transplant population.’^31^

Now to the clinical. Nigerian clinicians have a paper on the relationships between ‘cardiovascular signs and neurological signs in asphyxiated neonates.’^32^ This paper is followed by several on sickle cell anemia: ‘the burden of iron overload among non-chronically blood transfused preschool children^33^; disease severity and folate status^34^; utilization and provider-related barriers to the use of hydroxyurea in the treatment of sickle cell disease patients,^35^ and psycho-social problems of adolescents with sickle-cell anaemia.^36^ The section ends with an interesting blood transfusion paper on the utility of ‘home-made’ reagent red blood cells for antibody screening during pre-transfusion compatibility testing in Uganda.^37^

We have several papers on injury: traumatic spinal cord injury and clinical complications;^38^ acute kidney injury among medical and surgical in-patients in Ghana;^39^ mortality and its determinants after fragility hip fractures from Egypt^40^; and risk factors for childhood injuries in Tanzania.^41^ ending with a study of sports medicine practices for athletes in Uganda.^42^

We bring you several papers on sexuality: a new grading system for female sexual dysfunction in Egyptian women^43^; and one on menstrual hygiene management among adolescent school girls in Nigeria.^44^ A case report on refractory convulsive syncope in pregnancy and Takayasu's arteritis^45^ ends that brief section on sexuality. Some reflections on mental health include: effects of black tea consumption on depression^46^; genetic influence of Apolipoprotein E gene ε2/ε3/ε4 isoforms on odds of mesial temporal lobe epilepsy^47^; and correlates of substance use among undergraduates in a low income country.^48^ A rare paper on ‘psychological ailments and their treatment protocols based on a study of Swati traditional healers in Mpumalanga Province, in South Africa concludes the mental health section.^49^

The remaining papers are on diverse issues such as availability of low vision services in Ghana;^50^ utilization and uptake of the UpToDate clinical decision support tool51 at Makerere University in Uganda^51^; hearing healthcare gaps in a semi-urban community in Nigeria^52^. A paper on ‘upper gastrointestinal endoscopy findings in Uganda^53^; together with one on nutrition education on BMI-for-age in Ghanaian school-aged children^54^; and factors contributing to obesity and overweigh in Morocco^55^ give a glimpse on some aspects of nutrition in the continent. The effect of call reminders, short message services (SMS) reminders, and SMS immunization facts on childhood routine vaccination timing and completion in Nigeria^56^ reminds us of the potential for innovative technology in the health sector in Africa. We end this reflection on the June 2021 issue of African Health Sciences on a high note: the ‘clinical description and mutational profile of a Moroccan series of patients with Rubinstein Taybi syndrome.’^57^.

We thank all our readers, authors, reviewers, our team of highly dedicated volunteers, collaborators, partners and colleagues for their immeasurable contribution to *African Health Sciences*: the home of truly free online publishing in Africa!
